# Crystal Structure Determination and Hirshfeld Analysis of a New Alternariol Packing Polymorph

**DOI:** 10.3390/cryst12050579

**Published:** 2022-04-21

**Authors:** Kelly L. Rue, Guodong Niu, Jun Li, Raphael G. Raptis

**Affiliations:** 1 Department of Chemistry and Biochemistry, College of Arts, Science & Education, Modesto A. Maidique Campus, Florida International University, Miami, FL 33199, USA; 2 Department of Biological Sciences, College of Arts, Science & Education, Modesto A. Maidique Campus, Florida International University, Miami, FL 33199, USA; 3 Biomolecular Sciences Institute, College of Arts, Science & Education, Modesto A. Maidique Campus, Florida International University, Miami, FL 33199, USA

**Keywords:** polymorph, Hirshfeld analysis, alternariol

## Abstract

A new polymorph of the mycotoxin alternariol is reported and characterized by single crystal X-ray diffraction. Structural data, Hirshfeld surface analysis, and 2D fingerprint plots are used to compare differences in the intermolecular interactions of the orthorhombic *Pca*2_1_ Form I (previously reported) and the monoclinic *P*2_1_/*c* Form II (herein reported). The polymorphs have small differences in planarity—7.55° and 2.19° between the terminal rings for Form I and Form II, respectively—that brings about significant differences in the crystal packing and O-H … H interactions.

## 1. Introduction

**Scheme 1.**
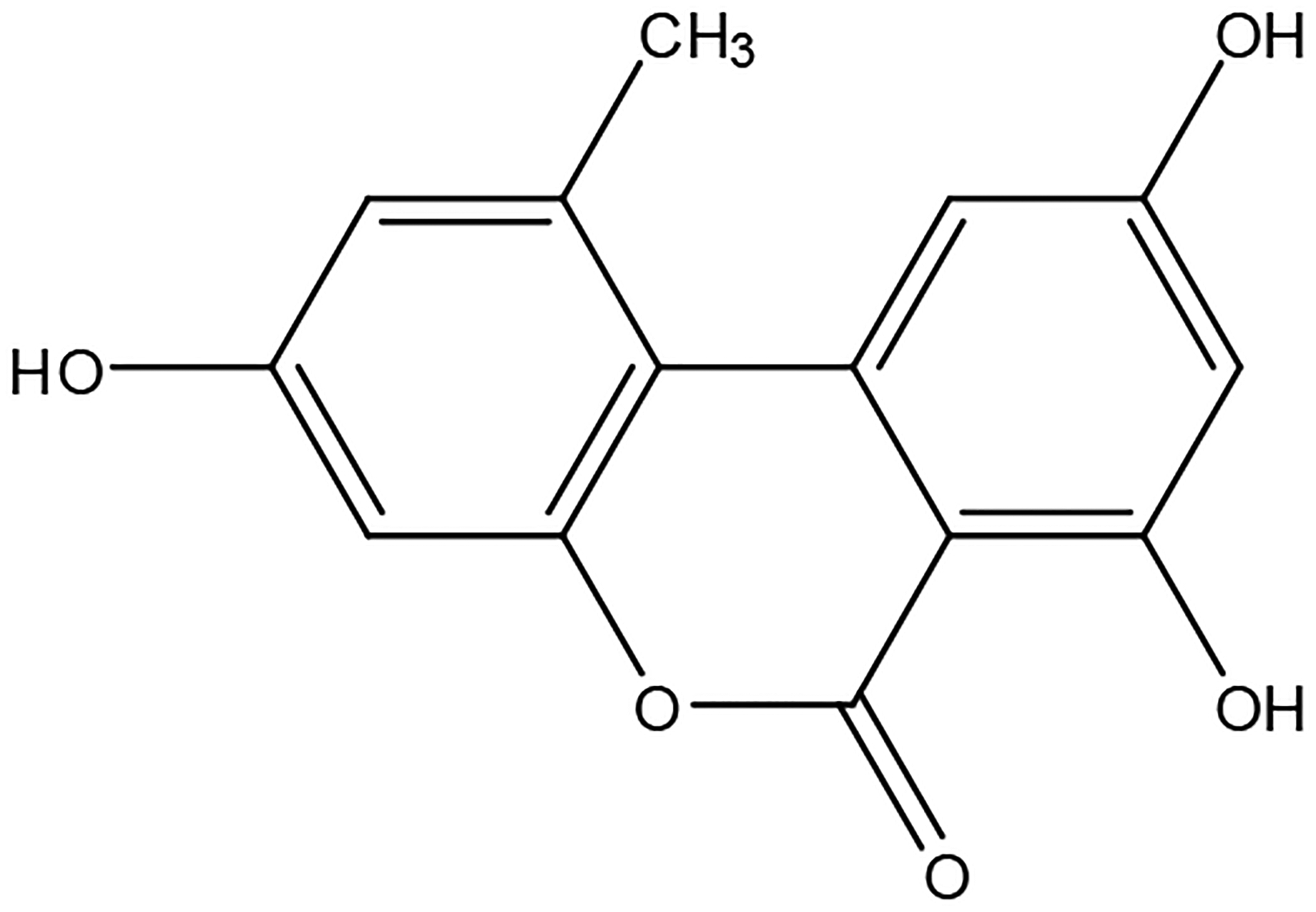
Line drawing of alternariol structure.

Alternariol (AOH; systematic name: 3,7,9-trihydroxy-1-methyl-6H-benzo[c]chromen-6-one; Scheme 1), a mycotoxin produced by various species of *Alternaria* molds, is an important contaminant in fruit, vegetable, and cereal products [1–3]. It possesses cytotoxic, genotoxic, and mutagenic properties in vitro [3–6]; however, these properties are still being studied in vivo. The underlying mechanism of toxicity is not yet fully established. AOH has been reported to induce the growth of the Alternaria species, *Alternaria alternata*, on various fruits [2,3].

Crystal polymorphs can exhibit different physical, chemical, and mechanical properties [7]. These are especially important for biologically active compounds, such as pharmaceuticals or mycotoxins [8–10]. The difference between polymorphic forms is in either the conformation or the packing arrangement of the molecules determining the intermolecular interactions [11–14]. An alternariol analogue, alternariol monomethyl ether (AME), with three reported structures in the Cambridge Crystallographic Data Centre (CCDC), exemplifies both polymorphism and solvomorphism (i.e., it is a compound which crystallizes in multiple space groups due to the presence of interstitial solvent molecules): two true polymorphs have crystallized in the *P*-1 and *Fdd*2 space groups, while the solvomorph has co-crystallized with dimethylsulfoxide in the *C*2/*m* space group [15–17].

In 2010, the crystal structure of AOH was published by Siegel et al. (CCDC refcode: TUPJOE), void of interstitial solvents; no polymorphs have been reported since [18]. Herein, we report the first polymorph of alternariol and discuss the differences in crystal structures and packing interactions. The previously reported structure will be identified as Form I and the one reported herein will be identified as Form II.

## 2. Materials and Methods

### 2.1. Materials

AOH was produced by the fungus *Purpureocillium lilacinum*, which was cultured on cereal. Secondary metabolites were extracted by ethyl acetate, and AOH was isolated by chromatography using the pulixin isolation procedure, as described previously [19]. All solvents were purchased from commercial sources and used without further purification.

### 2.2. Crystal Synthesis

Approximately 20 mg HPLC-pure AOH was dissolved in 2 mL methanol, and the solvent was allowed to evaporate at room temperature through two needle holes on the cover of the glass bottle. The colorless crystal used in the X-ray diffraction experiment was obtained on day three.

### 2.3. X-ray Crystallography and Data Collection

The slow evaporation of methanol under ambient conditions afforded colorless crystals of Form II. A suitable crystal was selected and mounted on a Bruker D8 Quest diffractometer equipped with a PHOTON 100 detector operating at T = 298 K (Bruker AXS, Madison, WI, USA). Data were collected with the shutterless ω-scan technique using graphite monochromated Mo-Kα radiation (λ = 0.71073 Å). The APEX3 [20] suite was used for collection, multiscan absorption corrections were applied, and structure solution was obtained using intrinsic phasing with SHELXT [21] (Bruker AXS, Madison, WI, USA). Data were then refined, using the Olex2 interface, by the least-squares method in SHELXL [22]. All hydrogen atoms were located in the difference map. Crystal data and structure refinement parameters are listed in Table 1. CCDC 2163068 contains the supplementary crystallographic data for this paper and can be obtained free of charge from The Cambridge Crystallographic Data Center via www.ccdc.cam.ac.uk/structures/. Hirshfeld surfaces were examined using *CrystalExplorer17* [23]. Interplanar geometric parameters were calculated using *Mercury 2020.3.0* [24].

### 2.4. Hirshfeld Surface Analysis

To illustrate differences in the intermolecular contacts of Form I and Form II, Hirshfeld surfaces were examined. Each surface has unique and well-defined points (*d*_*i*_, *d*_*e*_) where *d*_*i*_ represents a distance from a point on the Hirshfeld surface to the nearest nucleus internal to the surface and *d*_*e*_ represents a distance from a point on the surface to the nearest nucleus external to the surface. These points, along with the van der Waals radii, are normalized (*d*_*norm*_) and mapped onto the three-dimensional (3D) Hirshfeld surface where red regions represent close contacts (shorter than the sum of van der Waals radii) and negative *d*_*norm*_ values, blue regions represent long contact (longer than the sum of van der Waals radii) and positive *d*_*norm*_ values, and white regions represent a *d*_*norm*_ value of 0 (i.e., the contact distance is equal to the sum of van der Waals radii) [25–28]. These points can also be incorporated into two-dimensional (2D) fingerprint plots in which data are binned into pairs (*d*_*i*_, *d*_*e*_). Each bin is colored from blue (few points) to green (moderate points) to red (many points), and each point on the plot represents a bin with a width of 0.1 Å.

## 3. Results and Discussion

### 3.1. Structure Description of Form II

**Figure 1.**
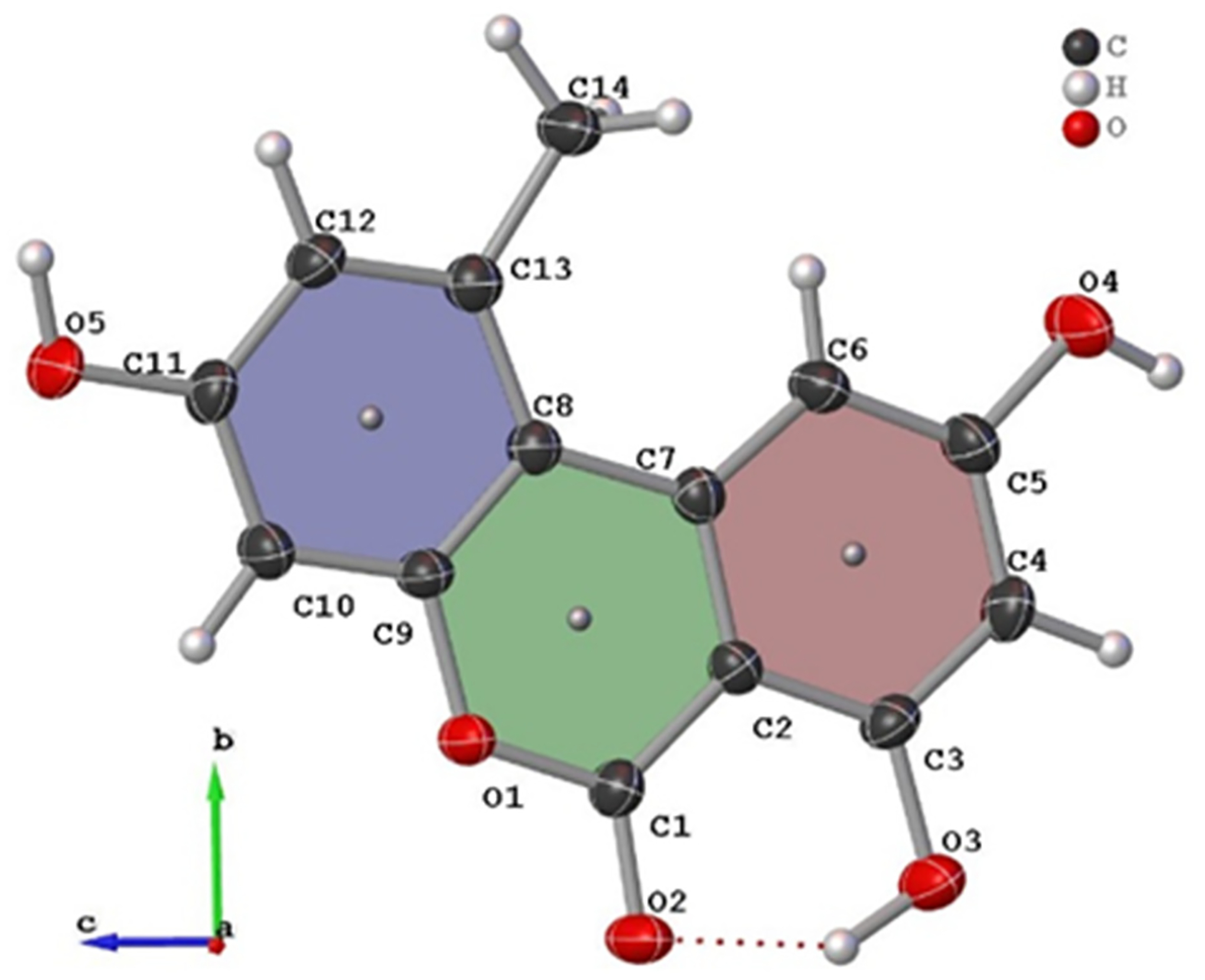
Crystal structure and labeling scheme of alternariol Form II with thermal ellipsoids at 50% probability. Plane 1 (red), Plane 2 (green) and Plane 3 (blue) are also shown.

**Figure 2.**
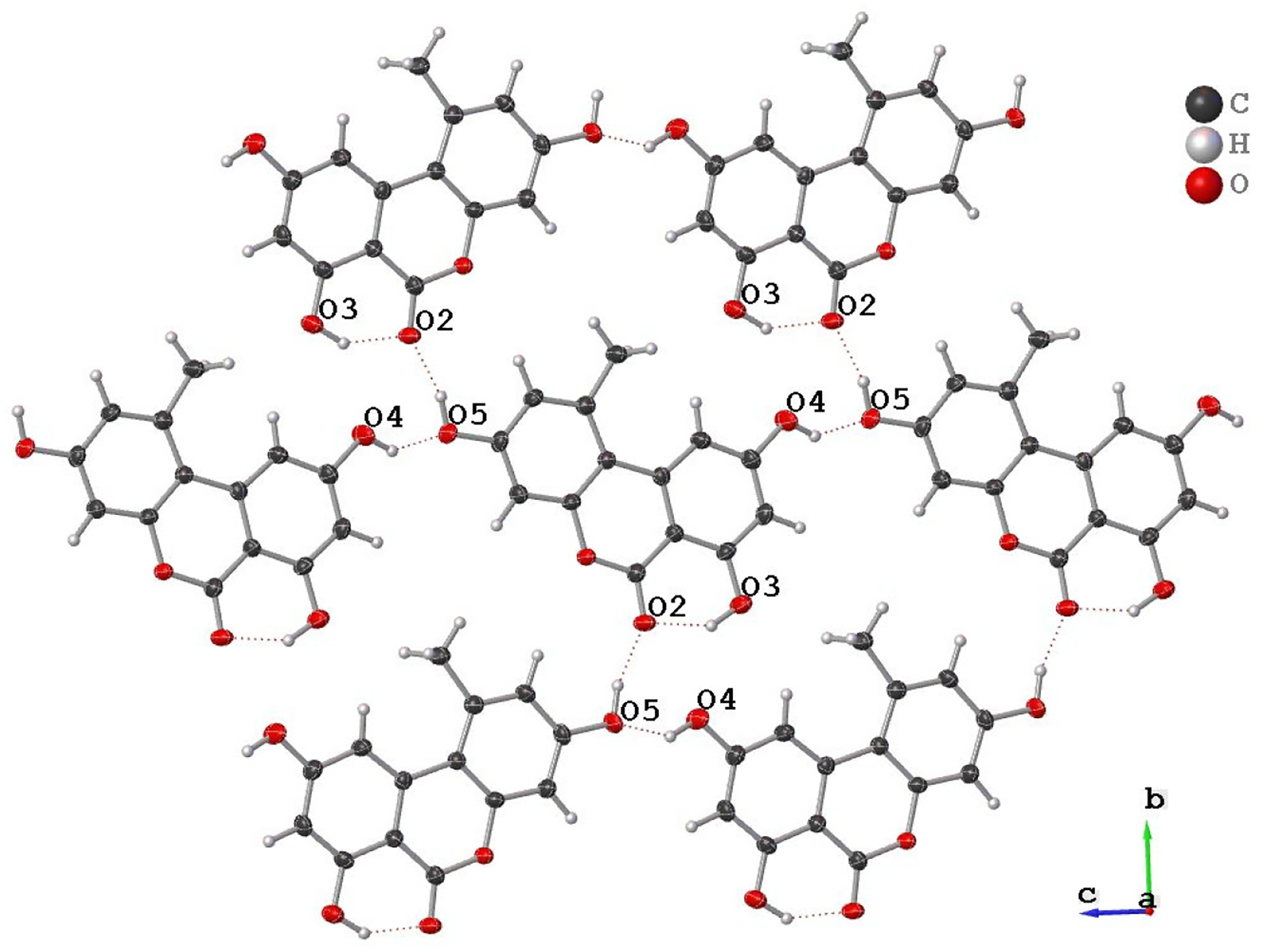
Hydrogen bonding network of alternariol—Form II. The middle molecule is surrounded by six other molecules and has classical H-bonding interactions with four of them.

**Figure 3.**
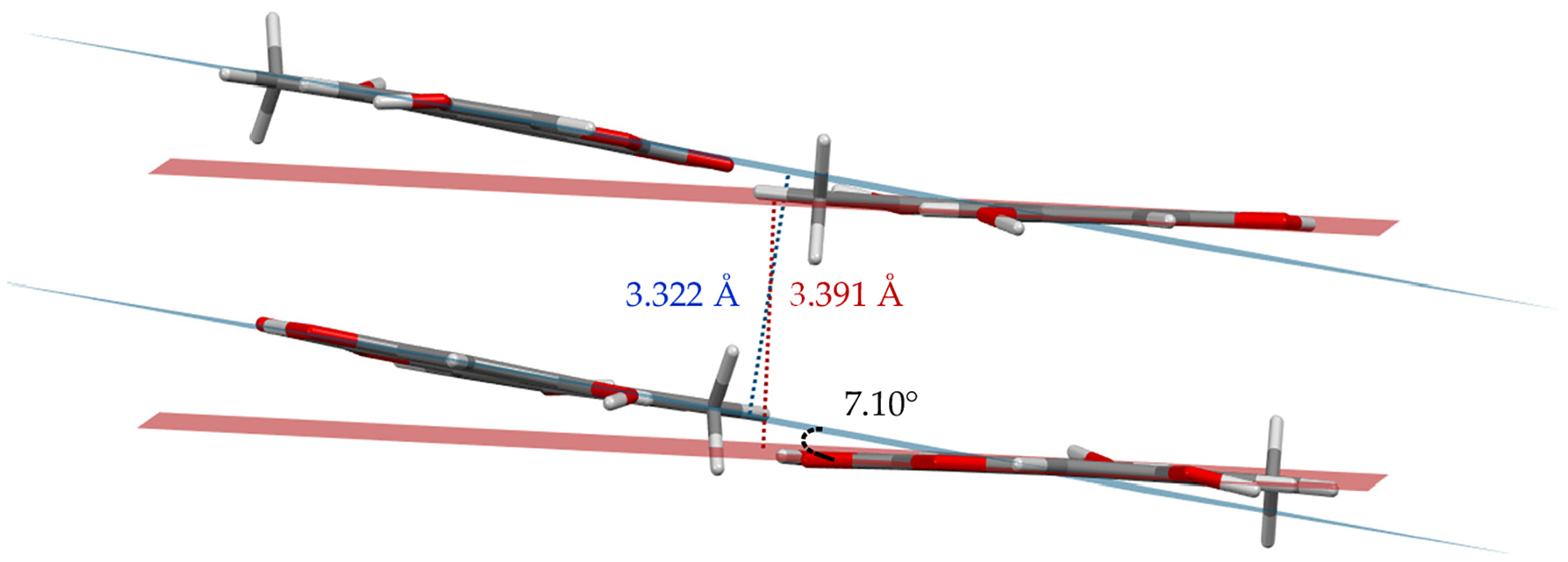
Perspective view depicting the interlayer distance and dihedral angle between best fit planes for two pairs of π-stacked molecules.

The new AOH polymorph (Form II) crystallized in the *P*2_1_/c space group. The molecule consists of three fused, six-membered rings (Ring 1: C2, C3, C4, C5, C6, C7; Ring 2: O1, C1, C2, C7, C8, C9; and Ring 3: C8, C9, C10, C11, C12, C13); the best-fit planes defined by these rings will be referred to as Plane 1, Plane 2, and Plane 3, respectively (Figure 1). The molecule is nearly planar with a plane twist angle of 2.19(8)° between Plane 1 and Plane 3 and surrounded by six approximately coplanar molecules. A strong intramolecular H-bond (O3-H … O2) is present in the molecular structure. A packing diagram (Figure 2) shows there are four additional intermolecular hydrogen H-bonds (O-H … O) per molecule (Table 2) and one non-classical hydrogen bonding interaction with a distance of 2.986(3) Å (O3-H … O4). Thus, each molecule has classical H-bonding interactions with four of the surrounding alternariol molecules and non-classical hydrogen bonding interactions with the remaining two molecules (Figure 2). All H-bonding interactions are between approximately coplanar molecules. There are two pairs of closely π-stacked layers with interlayer distances of 3.391 Å and 3.322 Å between each pair, while alternating pairs form a dihedral angle of 7.10° (Figure 3).

### 3.2. Comparison of Form I and Form II

Crystals of Form I were obtained by Siegel et al. via sublimation in an argon atmosphere and crystallized in the orthorhombic *Pca*2_1_ space group, while crystals of Form II were obtained via slow evaporation of a methanolic solution, crystallizing in the monoclinic *P*2_1_/*c* space group. There are three rotatable hydroxyl groups in alternariol, giving rise to eight possible conformations (as optimized by Scharkoi et al.) [29]; however, Forms I and II crystallize in the same conformation, which is different than the calculated gas phase energy minimum [29,30].

The rings of Form I are not strictly coplanar—there is a plane twist angle of 7.6(1)° between Plane 1 and Plane 3. Initially, the lack of planarity was hypothesized to be due to the steric effects of the methyl group (C14) in relation to the hydrogen atom on C6 (H6A). A benzo[c]chromen-6-one analogue, 2-chloro-7-hydroxy-8-methyl-6H-benzo[c]chromen-6-one [31], which lacks a sterically incumbered methyl group, yet its rings are considered to be coplanar with a plane twist angle of 2.78(8)°, was used for justification. However, this analogue also lacks intermolecular hydrogen bonding that may exert forces onto the hydroxyl groups, causing their respective rings to twist slightly out of plane. Form II exhibits an even smaller plane twist angle of 2.19(8)° than the above reference analogue. Moreover, the distance between the methyl group and H6A are statistically the same—2.36(3) Å and 2.39(2) Å for Form I and II, respectively. Therefore, the lack of planarity in Form I must be due to factors other than the steric interaction between the methyl group and H6A. A previously mentioned alternariol analogue, AME [15], solidifies this notion: like alternariol, it has a methyl group in close proximity to a hydrogen atom and a plane twist angle of only 0.59(5)°, suggesting that rings 1 and 3 are coplanar. The difference is that AME has a more extended hydrogen bonding network (including C-H … O and O-H … O interactions) and stronger π-π stacking.

**Figure 4.**
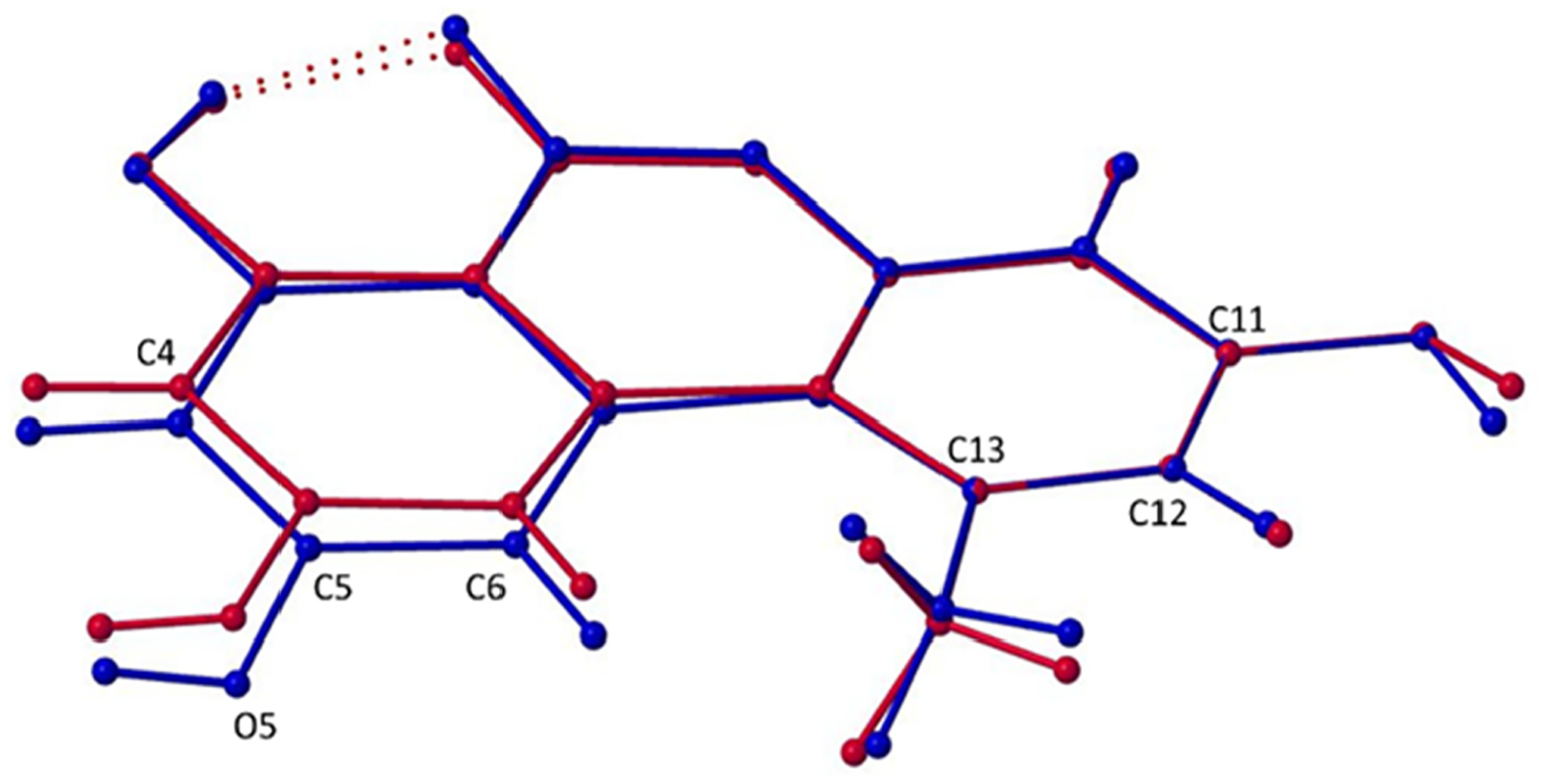
Overlayed wireframe images of alternariol Form I (blue) and Form II (red) matching C11, C12, and C13 of each structure.

**Figure 5.**
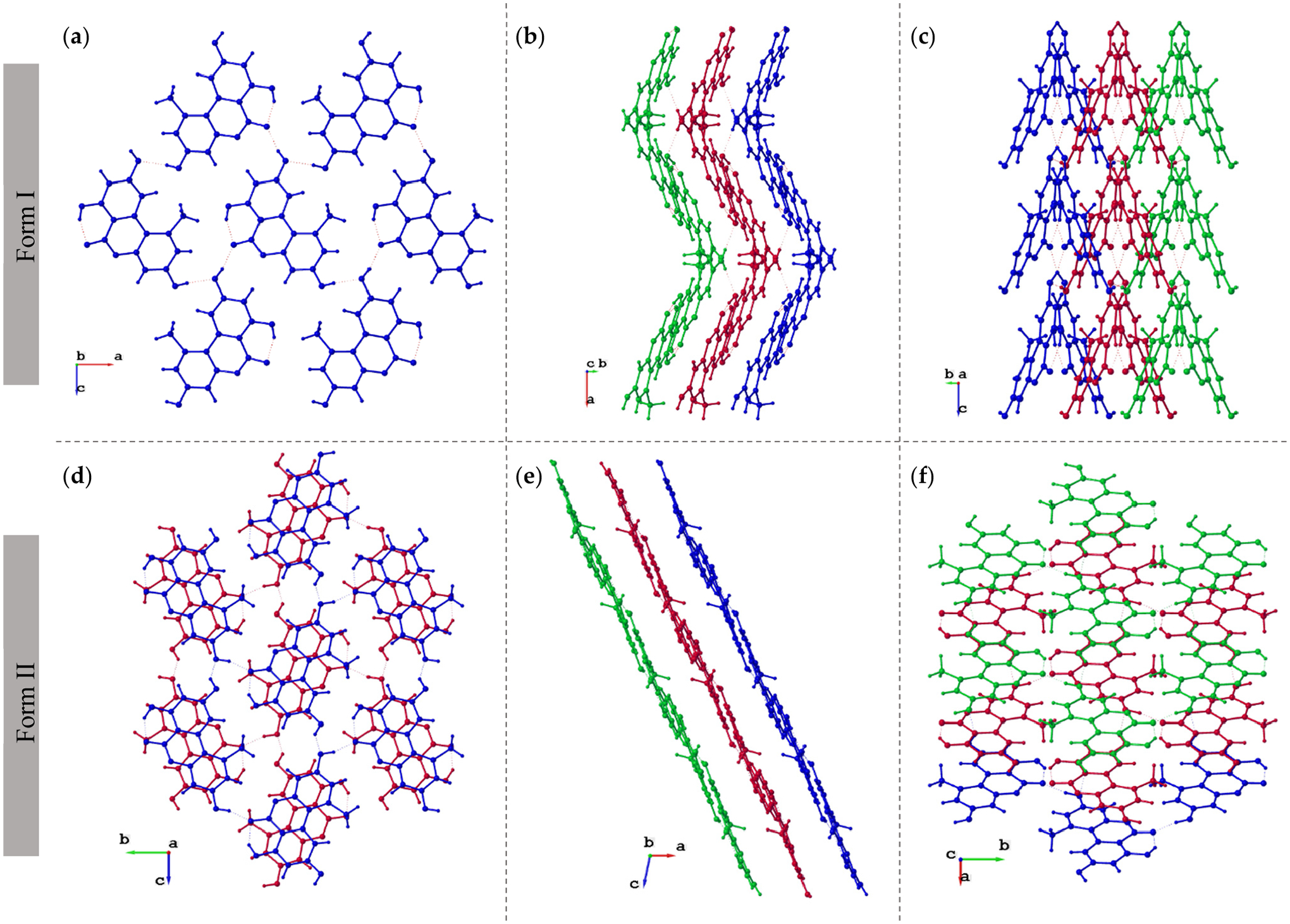
Crystal packing diagrams of Form I (upper) and Form II (lower) at different orientations. Left column (**a**,**d**): views perpendicular to molecular planes; Middle column (**b**,**e**): side views of the molecule; Right column (**c**,**f**): the same layers of **5b** and **5e** rotated by 90°.

Superimposed images of Form I and Form II, matching atoms C11, C12, and C13 of each structure (Figure 4), allow for visualization of the maximal deviations between the polymorphs occurring at C4, C5, O4, and C6 with differences of 0.256(3) Å, 0.352(4) Å, 0.503(3) Å, and 0.306(4) Å, respectively. Clearly, forms I and II of alternariol do not show significant variance in conformation and can therefore be described as packing polymorphs. The different packing arrangements are attributed to a slight difference in planarity between the two forms (Figure 5). In Form I, parallel molecules from different layers are eclipsed (Figure 5a) while in Form II adjacent molecules are rotated 180° and offset by approximately 1.46 Å. (Figure 5d). Form I exhibits a zig-zag packing motif where interlayer H-bonding interactions are formed between adjacent layers (Figure 5b), while Form II forms parallel layers (Figure 5e) with no interlayer H-bonding interactions.

#### Hirshfeld Surface Analysis

**Figure 6.**
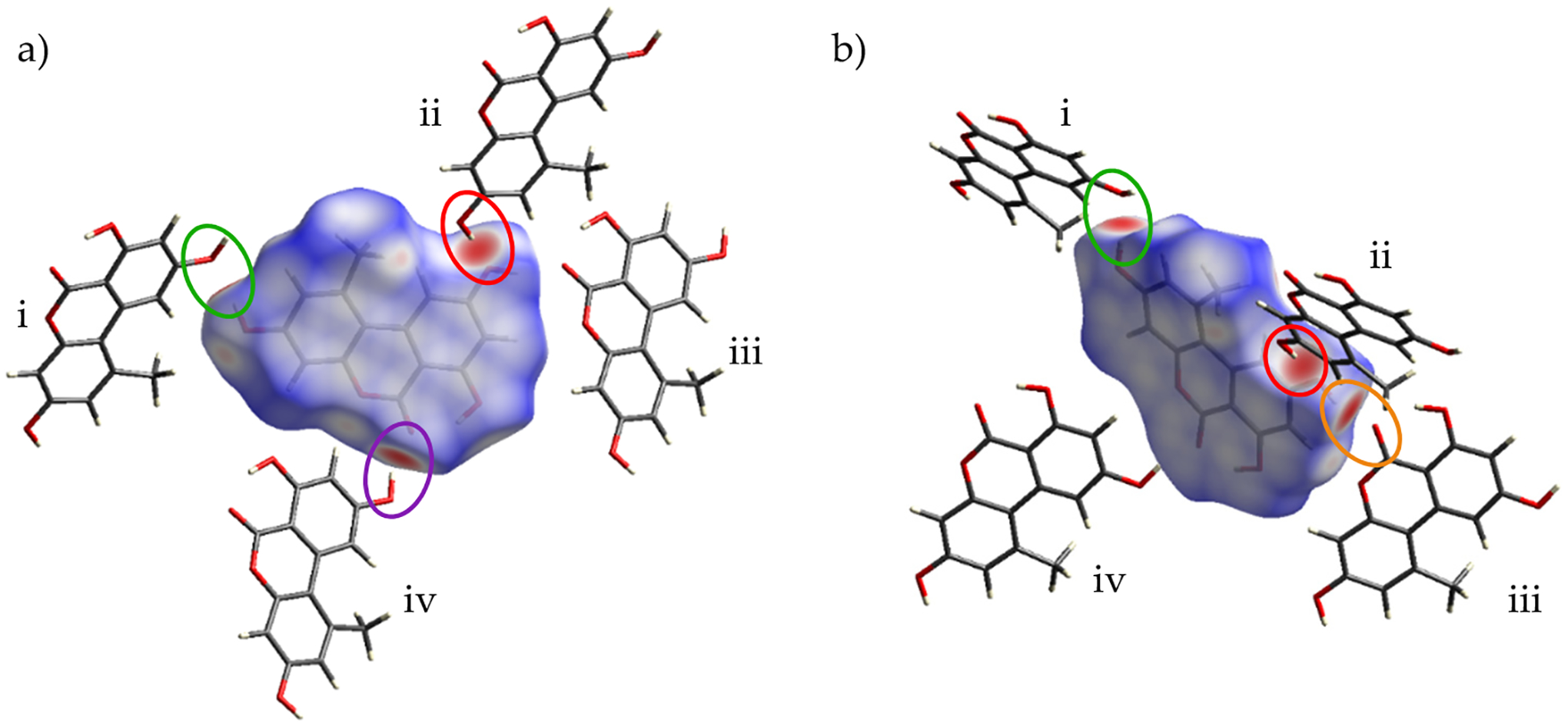
Hirshfeld surface of Form I mapped over d_norm_ along with external molecules i–iv: a perpendicular view of the molecule and its surface (**a**) and an angled view (**b**). Red regions on the surface represent close contacts, blue regions represent long contacts, and white regions represent contacts in which the distance is equal to the sum of van der Waals radii.

**Figure 7.**
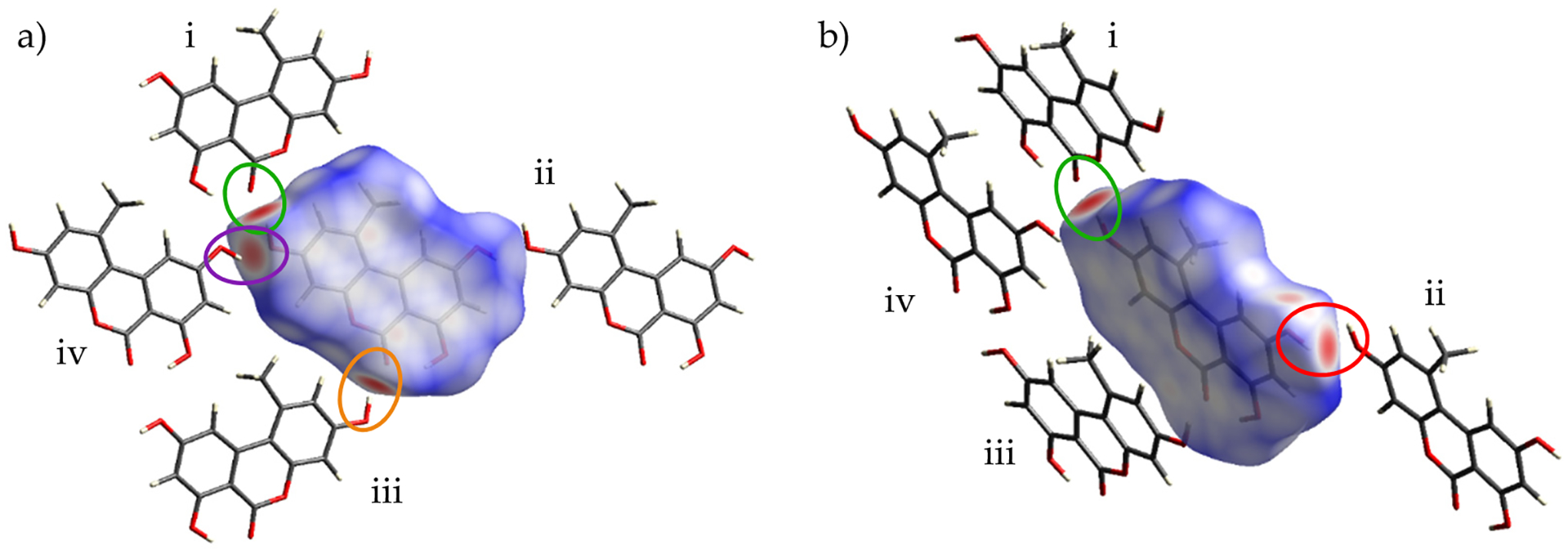
Hirshfeld surface of Form II mapped over d_norm_ along with external molecules i–iv: a perpendicular view of the molecule and its surface (**a**) and an angled view (**b**). Red regions on the surface represent close contacts, blue regions represent long contacts, and white regions represent contacts in which the distance is equal to the van der Waals radii.

Intermolecular interactions were investigated for Form I and Form II of alternariol via analysis of Hirshfeld surfaces. Figure 6 shows the Hirshfeld surface for Form I, mapped over *d*_*norm*_ (from −0.6764 to 1.0428) along with the neighboring molecules associated with the closest contacts. Figure 7 shows the Hirshfeld surface for Form II, mapped over *d*_*norm*_ (from −0.7193 to 1.2040) along with the neighboring molecules associated with the closest contacts. Figures 6a and 7a illustrate the contact points. In both figures, the red regions on the surface represent the closest interactions between molecules. Four intermolecular H-bonded interactions, O5-H … O4^i^ (2.809(3) Å; green), the reciprocal O4 … H-O5^ii^ (2.809(3) Å; red), O4-H … O2^iii^ (2.685(3) Å; orange), and O2 … H-O4^iv^ (2.685(3) Å; purple) dominate Form I (Figure 6). Similarly, four intermolecular H-bonded contacts are encountered in Form II: O5-H … O2^i^ (2.640(2) Å; green); O5 … H-O4^iv^ (2.735(2) Å; purple); O4-H … O5^ii^ (2.735(2) Å; red); and O2 … H-O5^iii^ (2.640(2) Å; orange) (Figure 7). In both Forms I and II, there is a pattern of two shorter, 2.685 Å (I) and 2.640 Å (II), and two longer, 2.809 Å (I) and 2.735 Å (II), intermolecular H-bonds, the ones of Form II being approximately 0.050 Å shorter than the corresponding ones in Form I. Counterintuitively, the tighter H-bonded pattern of Form II does not render it denser than I, with calculated densities of 1.594 g cm^−3^ vs. 1.590 g cm^−3^ for I and II, respectively. In Form I, three smaller, less intense red spots can be observed, denoting long-range interactions: the first one between the methyl group (C14) of external molecule i and O5 of the central molecule with a distance of 3.768(3) Å. The second long range interaction can be seen between the methyl group of the central molecule and the methyl group of an external one directly above it (not shown in Figure 5) with a distance of 3.7244 (6) Å. The third long range interaction at 3.547(3) Å can be seen between C4 of external molecule iv and O1 of the central one. This last interaction can also be seen between C4 … O1^iii^. A number of long-range interactions can also be seen in Form II. The first is a non-classical hydrogen bond between O4 of the central molecule with O3-H of an external one (shown in Figure 2) with a distance of 2.986(3) Å. The second interaction is between O1 of external molecule i and C12 of the central one with a distance of 3.821(3) Å. Two additional reciprocal interactions between C4 and the methyl group are shown at 4.126(3) Å.

**Figure 8.**
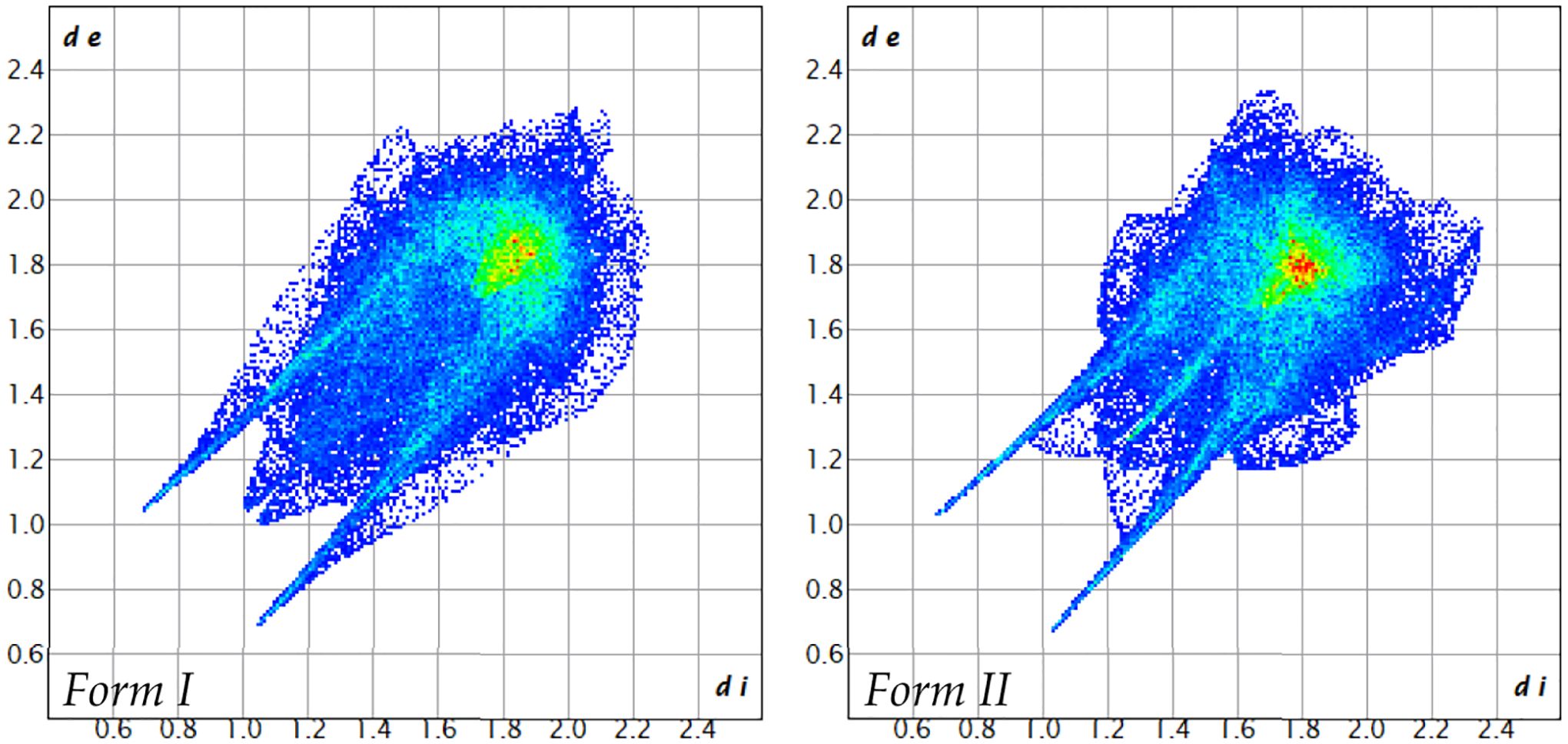
Full 2D fingerprint plot of Form I (**left**) and Form II (**right**) of alternariol.

From the *d*_*norm*_ surface, 2D fingerprint plots are assembled in Figure 8. Here, dark blue squares represent the fewest concentration of points, green—moderate, and red—dense concentration of points. The shapes of the full fingerprint plots for Forms I and II share some similarities. They each have two sharp spikes in the bottom left quadrant of the plot that correspond to the shortest interactions (O … H); however, the spikes of Form II are much sharper. Both plots also show a cluster of green and red squares roughly around *d*_*i*_ = *d*_*e*_ ≈ 1.8–2.0 which indicates C … C interactions and π-π stacking interactions; however, Form II has a larger concentration of these interactions.

The decomposition of the full fingerprint plot into the specific types of molecular interactions are shown in the Supplementary Materials. From the greatest to least contribution, the interactions are as follows (percent contributions are written in parentheses in the order of Form I, Form II): O … H (38.6, 37.4); H … H (28.9, 29.6); C … C (16.0, 16.2); C … H (9.1, 10.1); O … C (5.7, 4.7); O … O (1.7, 2.0). The order of contributions is the same for both Forms I and II; even the precent contributions of each interaction are similar for each Form.

## 4. Conclusions

Single crystals of two polymorphs of alternariol have been grown using two different methods—sublimation and recrystallization from a methanolic solution. As the bioavailability of polymorphs, and consequently their biological properties, vary among them, the possibility exists that recrystallization from solvents of a different polarity than methanol may result in additional polymorphic structures, which may exhibit differences in cytotoxicity.

## Supplementary Material

Supplemental File 1

## Figures and Tables

**Table 1.** Crystal data and structure refinement parameters for alternariol—Form II.

Formula	C_14_H_10_O_5_
D_calc._/g cm^−3^	1.590
μ/mm^−1^	0.122
Formula Weight	258.22
Color	Colorless
Shape	Plate
T/K	298
Crystal System	Monoclinic
Space group	*P*2_1_/c
a/Å	7.2836(3)
b/Å	14.3875(5)
c/Å	10.5110(3)
β/°	101.621(1)
V/Å^3^	1078.90(7)
Z	4
Wavelength/Å	0.71073
Radiation Type	Mo-K*α*
2θ_min_/°	4.8
2θ_max_/°	52.8
Measured Refl.	23,218
Independent Refl.	2213
Reflections Used, I_o_ > 2s(I_o_)	1435
R_int_	0.083
Parameters	213
^a^ GooF	1.021
^b^ wR_2_	0.1177
^c^ R_1_	0.0505

a $\text{GooF}={\left[\sum \left[w{\left(F_{o}^{2}-F_{c}^{2}\right)}^{2}\right]/\left(N_{o}-N_{v}\right)\right]}^{1/2}$;

b ${\text{wR}}_{2}=\sum \left|\left|F_{o}\right|-\left|F_{c}\right|\right|/\sum \left|F_{o}\right|$;

c ${\text{R}}_{1}={\left[\left(\sum w{\left(F_{o}^{2}-F_{c}^{2}\right)}^{2}/\sum {\left|F_{o}\right|}^{2}\right)\right]}^{1/2}$.

**Table 2.** Hydrogen-bond geometry (Å, °) of alternariol—Form II.

*D*-H … *A*	*D*-H	H … *A*	*D* … *A*	*D*-H … *A*
O3-H3 … O2	0.94(4)	1.76(4)	2.590(2)	144(3)
O4-H4 … O5^i^	0.89(3)	1.99(3)	2.735(2)	140(3)
O5-H5 … O2^ii^	0.93(3)	1.74(3)	2.640(2)	163(3)
O3-H3 … O4^iii^	0.94(4)	2.34(3)	2.986(3)	125(3)

i *x* − 1, *y*, *z* − 1;

ii −*x* + 1, *y* + 1/2, −*z* + 3/2;

iii −*x*, *y* − 1/2, −*z* + 1/2.
