# Supplementary material for: Crystal Structure Determination and Hirshfeld Analysis of a New Alternariol Packing Polymorph
Source: Crystals (Basel). Author manuscript; Available in PMC 2022 Aug 12. (PMC9374539; doi:10.3390/cryst12050579)
Supplement: Supplemental File 1 [file NIHMS1828904-supplement-Supplemental_File_1.zip › crystals-1685804-supplementary.pdf]

Supplmentary Information

Crystal structure determination and Hirshfeld analysis of a new alternariol packing polymorph

Kelly Rue[1], Guodong Niu[2], Jun Li[2,3,*], Raphael Raptis[1,3,*]

[1] Department of Chemistry and Biochemistry, Florida International University, Miami, FL 33199, USA

[2] Department of Biological Sciences, Florida International University, Miami, FL, 33199, USA

[3] Biomolecular Sciences Institute, Florida International University, Miami, FL, 33199, USA

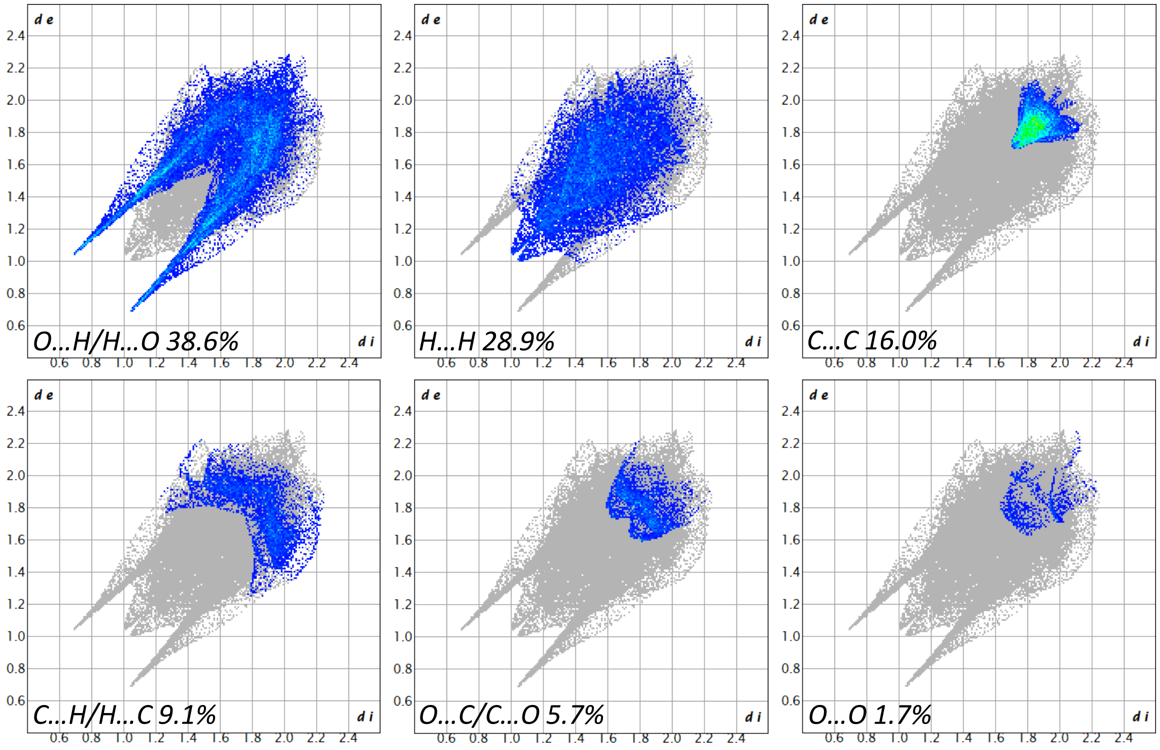

Figure S1. 2D fingerprint plots filtered by type of molecular interaction of alternariol – Form I.

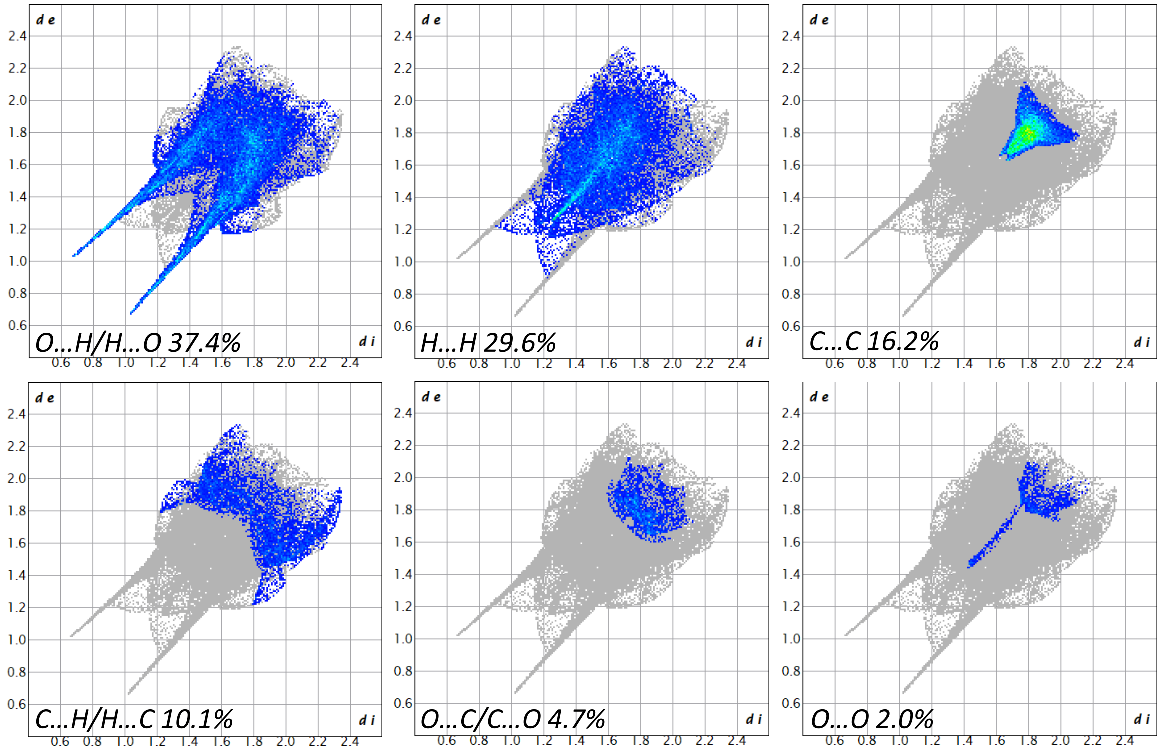

Figure S2. 2D fingerprint plots filtered by type of molecular interaction alternariol – Form II.

Table S1. Selected bond lengths (Å) for alternariol – Form II

O1—C9	1.387 (3)	C7—C6	1.384 (3)
O1—C1	1.346 (3)	C11—C10	1.371 (3)
O3—C3	1.346 (3)	C11—C12	1.389 (3)
O3—H3	0.94 (4)	C11—O5	1.360 (3)
O2—C1	1.231 (3)	C3—C2	1.412 (3)
O4—C5	1.357 (3)	C3—C4	1.380 (3)
O4—H4	0.89 (3)	C13—C12	1.378 (3)
C9—C8	1.397 (3)	C13—C14	1.505 (3)
C9—C10	1.378 (3)	C2—C1	1.426 (3)
C8—C7	1.474 (3)	C4—C5	1.373 (3)
C8—C13	1.430 (3)	C5—C6	1.388 (3)
C7—C2	1.425 (3)	O5—H5	0.93 (3)
